# Facilitating Neuron-Specific Genetic Manipulations in *Drosophila melanogaster* Using a Split GAL4 Repressor

**DOI:** 10.1534/genetics.116.199687

**Published:** 2017-03-29

**Authors:** Michael-John Dolan, Haojiang Luan, William C. Shropshire, Ben Sutcliffe, Benjamin Cocanougher, Robert L. Scott, Shahar Frechter, Marta Zlatic, Gregory S. X. E. Jefferis, Benjamin H. White

**Affiliations:** * Division of Neurobiology, Medical Research Council Laboratory of Molecular Biology, Cambridge Biomedical Campus, CB2 0QH, United Kingdom; † Janelia Research Campus, Howard Hughes Medical Institute, Ashburn, Virginia 20147; ‡ Laboratory of Molecular Biology, National Institute of Mental Health, National Institutes of Health, Bethesda, Maryland 20892

**Keywords:** *Drosophila*, Gal4-UAS, LexA-LexAop, transgene expression, neural circuits

## Abstract

Efforts to map neural circuits have been galvanized by the development of genetic technologies that permit the manipulation of targeted sets of neurons in the brains of freely behaving animals. The success of these efforts relies on the experimenter’s ability to target arbitrarily small subsets of neurons for manipulation, but such specificity of targeting cannot routinely be achieved using existing methods. In *Drosophila melanogaster*, a widely-used technique for refined cell type-specific manipulation is the Split GAL4 system, which augments the targeting specificity of the binary GAL4-UAS (Upstream Activating Sequence) system by making GAL4 transcriptional activity contingent upon two enhancers, rather than one. To permit more refined targeting, we introduce here the “Killer Zipper” (KZip^+^), a suppressor that makes Split GAL4 targeting contingent upon a third enhancer. KZip^+^ acts by disrupting both the formation and activity of Split GAL4 heterodimers, and we show how this added layer of control can be used to selectively remove unwanted cells from a Split GAL4 expression pattern or to subtract neurons of interest from a pattern to determine their requirement in generating a given phenotype. To facilitate application of the KZip^+^ technology, we have developed a versatile set of LexA_op_-KZip^+^ fly lines that can be used directly with the large number of LexA driver lines with known expression patterns. KZip^+^ significantly sharpens the precision of neuronal genetic control available in *Drosophila* and may be extended to other organisms where Split GAL4-like systems are used.

ANIMAL nervous systems consist of large numbers of highly heterogeneous cells that often differ profoundly in function (Luo *et al.* 2008). This functional heterogeneity represents a major challenge to understanding how nervous systems work because it requires analyzing the biological roles of not one, or a few, neural cell types, but many. In the extreme case, each individual neuron in an animal might need to be selectively interrogated to determine its contribution to behavior or other brain processes. Although routine analysis at this level is not currently feasible except in animals with extremely reduced nervous systems, it is increasingly possible to target populations of neurons—often of a particular type—for functional interrogation using genetic methods. Such methods have been developed to facilitate neural circuit mapping in genetic model organisms and have been used successfully for that purpose in mice (Kitamura *et al.* 2014; Li *et al.* 2015), fish (Lacoste *et al.* 2015), and fruit flies (Dubnau *et al.* 2001; Claridge-Chang *et al.* 2009). Nevertheless, the number of circuits that have yielded to this approach, and the resolution to which they have been mapped, has been constrained by the limited ability of existing methods to systematically and reproducibly target small, cell type-specific neuronal populations. This is true even in the fruit fly, *Drosophila melanogaster*, where currently available techniques can sometimes achieve single-cell resolution.

Most methods for the selective manipulation of cellular function in *Drosophila* build on the binary GAL4-UAS (Upstream Activating Sequence) system (Brand and Perrimon 1993; Pfeiffer *et al.* 2010). In this system, expression of the GAL4 transcription factor is directed to specific cells using an enhancer element of an endogenously expressed gene. Within the targeted cells, GAL4 activates expression of transgenes placed under the control of its UAS and the products of these transgenes then alter neuronal function. While the GAL4-UAS system has occasionally been successful in targeting small groups of neurons, its targeting specificity is, in general, constrained by the limited cell type selectivity of the neuronal gene enhancers required to drive expression of GAL4 (Pfeiffer *et al.* 2008; Jenett *et al.* 2012).

To provide greater selectivity of targeting, several ternary expression systems have been developed that effectively restrict the activity of GAL4 to a population of neurons in which two gene enhancers, rather than one, are required to be active (Luan *et al.* 2006; Bohm *et al.* 2010; Potter *et al.* 2010; Ting *et al.* 2011). This combinatorial strategy is the basis of the Split GAL4 system, which is widely used for cell type-restricted expression in the fly (Luan *et al.* 2006; Pfeiffer *et al.* 2010). In this system, the two enhancer elements are used to independently target the GAL4 DNA-binding domain and a complementary transcription activation domain to different cell groups. Only in cells in which both enhancers are active are the two transcription factor domains coexpressed. Each domain is fused to one of a pair of heterodimerizing leucine zippers (Zip^–^ and Zip^+^), which allows them to come together in coexpressing cells to reconstitute transcriptional activity and drive the expression of UAS-transgenes.

The refinement of transgene expression afforded by the Split GAL4 system has facilitated the targeting of single neuronal cell types in an increasing number of cases (Luan *et al.* 2012; Kohl *et al.* 2013; Tuthill *et al.* 2013; Aso *et al.* 2014a,b; Bidaye *et al.* 2014; Diao *et al.* 2015; Hoopfer *et al.* 2015). However, this exquisite precision has proved difficult to achieve consistently even using two enhancers. To improve the targeting specificity of the Split GAL4 system, we here introduce an additional mechanism of transcriptional control similar to that provided by GAL80 in the binary GAL4-UAS system (Lee and Luo 1999; Suster *et al.* 2004). We have developed the “Killer Zipper” (KZip^+^), a dominant-negative repressor of Split-GAL4 activity, which can be expressed under the control of a third enhancer to exclude defined cells from a Split GAL4 expression pattern. We validate the efficacy of KZip^+^ constructs and demonstrate their use in the refinement of Split GAL4 expression in the fly brain.

## Materials and Methods

### Molecular biology

All molecular biology was performed following standard procedures (Maniatis *et al.* 2012) and either Gibson Isothermal Assembly (Gibson *et al.* 2009) or Gateway Cloning (Invitrogen, Carlsbad, CA). One Shot Mach1 T1 Phage-Resistant Chemically Competent *Escherichia coli* (Life Technologies) or *Mix & Go* Competent Cells - Strain Zymo 5α (Zymo Research, Irvine, CA) were used for chemical transformations. DNA was prepared using QIAprep Spin Miniprep or Midiprep Kits (QIAGEN, Valencia, CA) and QIAquick Gel Extraction, and PCR Purification Kits (QIAGEN) or Zymoclean Gel DNA Recovery Kit (Zymo Research) were used to extract or purify DNA fragments, respectively. All restriction enzymes were purchased from New England Biolabs (NEB) (Beverly, MA). Gibson Isothermal reaction mix was either purchased (NEB) or created from a published recipe (Gibson *et al.* 2009). All plasmids were sequence-verified and sequencing services were provided by GATC (GATC Biotech AG, Konstanz, Germany), Source Biosciences (Nottingham, UK) or Macrogen (Rockville, MD). Oligonucleotide synthesis was carried out by Integrated DNA Technologies (Coralville, IA) and gene synthesis by Epoch Life Science (Sugar Land, TX).

#### CCAP-KZip^+^ and Burs-KZip^+^ constructs:

KZip^+^ was made from previously published components as described in Luan *et al.* (2006) and placed behind CCAP (Crustacean Cardioactive Peptide) and bursicon promoters (Burs) in the pCaST X11 *P*-element transformation vector. Briefly, the Zip^−^ (*i.e.*, RR_12_EE_345_L) moiety of the Zip^−^-GAL4DBD was replaced by the Zip^+^ (*i.e.*, EE_12_RR_345_L) moiety previously used to make the GAL4AD-Zip^+^ and VP16AD-Zip^+^ constructs. The resulting KZip^+^ construct was inserted into the pCaST-CCAP-GAL4DBD vector using flanking *Not*I and *Asc*I restriction sites to replace Zip^−^-GAL4DBD. This pCaST-CCAP-KZip^+^ vector was then used to make pCaST-Burs-KZip ^+^ by replacing the CCAP promoter with the previously described promoter of the *Burs* (*i.e.*, *bursicon* α) gene (Peabody *et al.* 2009) using flanking *Eco*RI and *Not*I restriction sites.

The five KZip^+^ variants expressed under the control of the CCAP promoter were prepared for ΦC31-mediated transgenesis in a vector derived from pJFRC81-10XUAS-IVS-Syn21-GFP-p10 (Pfeiffer *et al.* 2012). First, the unaltered CCAP-KZip^+^ variant was made by replacing the 10XUAS-IVS-Syn21-GFP-p10 sequence of this vector with the CCAP-KZip^+^ sequence from pCaST-CCAP-KZip^+^ together with the Simian virus 40 transcription terminator. The sequence encoding KZip^+^ in this vector is flanked by unique *Eag*I and *Xba*I restriction sites, which allowed it to be replaced by similarly flanked sequences encoding 4xKZip^+^, KZip^+^::HRD, and KZip^+^::3xHA, respectively. The latter sequences were synthesized by Epoch Life Science (Sugar Land, TX) and contain, in order: a construct consisting of four tandem copies of KZip^+^ separated by viral T2A peptides; a C-terminal fusion of KZip^+^ to the Hairy Repressor Domain, an 18 aa peptide (*i.e.*, Hairy aa320–337) shown to repress transcriptional activity (Fisher *et al.* 1996); and a C-terminal fusion of KZip^+^ to three copies of the hemagglutinin epitope tag. A final variant, CCAP-IVS-Syn21-KZip^+^-p10, retains the viral translational enhancers of pJFRC81-10XUAS-IVS-Syn21-GFP-p10 and was derived from that vector by replacing the 10XUAS sequence with that of the CCAP promoter and the coding sequence of GFP with that of the KZip^+^ construct.

#### LexA_op_-KZip^+^ constructs:

All LexA_op_-KZip^+^ constructs were made in a vector derived from pJFRC19 (Pfeiffer *et al.* 2010). LexA_op_-KZip^+^::3xHA was made by amplifying KZip^+^ from pCaST-CCAP-KZip^+^ using PCR primers that also incorporated a 3xHA-tag at the C-terminus (pJFRC19/KZ_F and pJFRC19/KZ::HA_R; Supplemental Material, Table S1). These primers contained overhanging regions identical to regions flanking the myrGFP ORF in pJFRC19 (Pfeiffer *et al.* 2010). The pJFRC19 plasmid backbone was amplified inversely without the myrGFP ORF (using pJFRC19inverse_F and pJFRC19inverse_R primers, Table S1). The linearized, pJFRC19 backbone was used for a Gibson Isothermal reaction with the KZip^+^::3xHA PCR product to produce pLexA_op_-KZip^+^::3xHA.

For the generation of all other constructs we switched to a digestion strategy, as the PCR amplification of large plasmid backbones rarely produced sufficient quantities for cloning. The pLexA_op_-KZip^+^::3xHA vector was used to make subsequent constructs by first excising the KZip^+^::3xHA ORF by restriction digestion with *Not*I-HF and *Xba*I, and then using the resulting backbone (purified by gel extraction) for Isothermal assembly together with a gel-extracted PCR product corresponding to the KZip^+^ construct. For LexA_op_-KZip^+^, the KZip^+^ PCR product was amplified from pLexA_op_- KZip^+^::3xHA using PCR primers that excluded the HA-tag (pJFRC19/KZip^+^_F and pJFRC19_Stop_ KZip^+^_R; Table S1). For nucLacZ-T2A-KZip^+^, the PCR product was amplified from a synthesized gene placed in the pMA vector (pMA-nucLacZ-T2A-KZip^+^; GeneArt, Thermo Fisher) using the pMA-LacZ_F and pMA- KZip^+^_R primers (Table S1). nucLacZ represents a fusion of the LacZ gene a nuclear targeting sequence and T2A is the 2A peptide from *Thosea asigna*, which promotes ribosomal skipping. By placing the LacZ gene before the T2A sequence we ensured minimal disruption of the KZip^+^ protein sequence, replacing only the N-terminal methionine with a proline residue.

### Fly stocks

All transgenic fly lines were created either by *P* element-mediated or ΦC31-mediated transgenesis via embryonic microinjection, which was performed by Rainbow Transgenic Flies (Camarillo, CA) or BestGene (Chino Hills, CA). The chromosomes (for *P*-element insertions) or sites (for ΦC31-mediated transgenesis) of insertion for the various lines made, as well as the constructs used, are as listed in Table S1. The CCAP-KZip^+^ and Burs-KZip^+^ made by *P*-element transgenesis were initially screened for efficacy by crossing to “tester lines” of the following general genotype: (y)w^1118^; UAS-Reporter/effector; elav-VP16AD^G3A1^, CCAP-GAL4DBD^K5A1^/TM6b, where the UAS-driven constructs were: 2XEGFP, 2XEKO, or NaChBac^B-16^. Expression of either 2XEKO or NaChBac^B-16^ has previously been shown to cause an unexpanded wing phenotype, and progeny were scored for suppression of this phenotype. Lines that gave high levels of suppression of either the unexpanded wing phenotype or enhanced GFP (EGFP) reporter expression when examined by fluorescence microscopy in CNS wholemounts, were selected for the experiments shown.

Previously described stocks used in this study include: yw, Shaw^MI01735^-p65AD (Diao *et al.* 2015), w; +; UAS-TRPM8^C4-A^ (Peabody *et al.* 2009) (note that TRPM8 in the C4-A stock is tagged with EGFP at the N-terminus, although this detail was unwittingly omitted in the original publication), w; +; elav-GAL4DBD^H4A1^ (Luan *et al.* 2006), w; +; CCAP-GAL4DBD (Luan *et al.* 2006), w;IF/CyO;Nsyb-LexA (VK00037) (Pfeiffer *et al.* 2010), yw,UAS-CD8::GFP;UAS-CD8::GFP;JK1029-AD,ChaDBD/TM6b (Kohl *et al.* 2013), w;if/Cyo;MB247-LexA/Tm6b (Pitman *et al.* 2011), w,Repo-LexA (Lai and Lee 2006), w;UAS-CD8::GFP/CyO (Lee and Luo 1999), MB112C: w;;(R93D10-p65ADZp (VK00027), R13F04-ZpGAL4DBD in attP2) (Aso *et al.* 2014a), and *teashirt*-LexA/Cyo;TM6b/MKRS (kindly provided by J.-M. Knapp and J. Simpson). In addition, two Split-GAL4 lines not previously described were used in this study. One (w;;JK801-VP16AD, SF131-DBD) expresses in a Lateral Horn cell type and in many glia throughout the brain. A second (w;126E12-ADp65;103H02-DBD) drives in the larval central brain and ventral nerve cord (VNC).

### Immunohistochemistry

Whole-mount immunohistochemistry was performed essentially as described before (Luan *et al.* 2012; Ostrovsky *et al.* 2013). The primary antibodies used were: 1:20 mouse anti-nc82 (DSHB, University of Iowa), 1/1600 chicken anti-GFP (Abcam, Cambridge, UK), 1/400 rabbit anti-HA (Cell Signaling Technology, Danvers, MA), 1/400 mouse anti-β-galactosidase (Abcam), 1:3000 rabbit anti-CCAP (Jena Bioscience, Jena, Germany), and 1:100 mouse anti-GFP (Thermo Fisher, Waltham, MA). The secondary antibodies used were all purchased from Life Technologies, now Thermo Fisher: Alexa-568 anti-mouse (for nc82 or β-galactosidase staining), Alexa-633 anti-mouse IgG1 (for nc82 staining), Alexa-488 anti-chicken (for anti-GFP or anti-mVenus staining), Alexa-488 anti-mouse (for anti-GFP staining), Alexa-568 anti-rabbit (for HA staining), and Alexa-555 anti-rabbit (for CCAP staining). All secondary antibodies were raised in goat and used at 1/1600 concentration, and all specimens were mounted in Vectashield (Vector Laboratories, Burlingame, CA).

In all experiments, control and experimental preparations (*i.e.*, ±KZip^+^) were processed entirely in parallel to permit direct comparison of the results. Also, except for the optimization experiments shown in Figure S1, UAS-reporter expression was amplified by immunostaining using anti-GFP to stringently test for suppression of Split GAL4 activity by KZip^+^. Anti-GFP immunostaining was not used in the optimization experiments in which it was important to accurately compare the efficacy of the five CCAP-KZip^+^ variants made by ΦC31-mediated transgenesis (Groth *et al.* 2004). To do so, the CNSs of newly eclosed flies were excised and mounted after fixation as described previously (Luan *et al.* 2012). All whole-mount preparations were imaged using a Nikon C-1 confocal microscope and a 20 × objective under the same conditions. To estimate the efficacy of each KZip^+^ variant in suppressing CCAP-GAL4DBD∩elav-VP16^AD^-driven expression of UAS-2XEGFP, we quantified the residual labeling of the CCAP-expressing neurons as follows: For each of 6–9 preparations imaged as described above, maximum intensity projections of the Z-stacks were examined and the intensity of EGFP signal for each labeled CCAP neuron cell body was scored on a scale of 1–3, with one being low-level labeling and three being high-level labeling. The average number of EGFP-positive CCAP neurons was then calculated for the CNS preparations of each genotype together with the average intensity of the labeled cells.

### Wing expansion assay

Flies were raised at 25° and analyzed immediately after eclosion for the time taken to expand their wings under conditions of confinement, as described previously (Peabody *et al.* 2009). Briefly, progeny from crosses listed below were immediately confined in a minichamber (Peabody *et al.* 2009) and then placed on a Peltier plate at 25° or 18° and videorecorded until wing expansion. Expansion time was measured as the time from eclosion until the wings had completely expanded and were folded over the back.

### Statistics

For all experiments, the number of observations is indicated in the figure legend. Each experiment was repeated at least twice with independent groups of flies.

For the wing expansion data in Figure 5, a Levene test indicated the data were heteroscedastic. The sample size for each experiment is indicated in the corresponding figure legend. No power analysis was performed to determine sample size, rather the sample sizes used were similar to those in previous work describing neurogenetic tools (Pfeiffer *et al.* 2010; Potter *et al.* 2010; Riabinina *et al.* 2015) and wing expansion experiments (Peabody *et al.* 2009; Luan *et al.* 2012). Significant differences between samples were demonstrated using a Welch’s One Way ANOVA (*P* < 2.2e–16), where *P* < 0.05 was declared significant. Three Welch *t*-tests were conducted between the temperature levels for each genotype and the *P*-values were adjusted using the Bonferroni correction. Outliers were not excluded from analysis.

### Data availability

KZip^+^ constructs and fly lines are available upon request. File S1 lists the genotypes of all animals used in this study by figure.

## Results

### Design of KZip^+^

**Figure 1:**
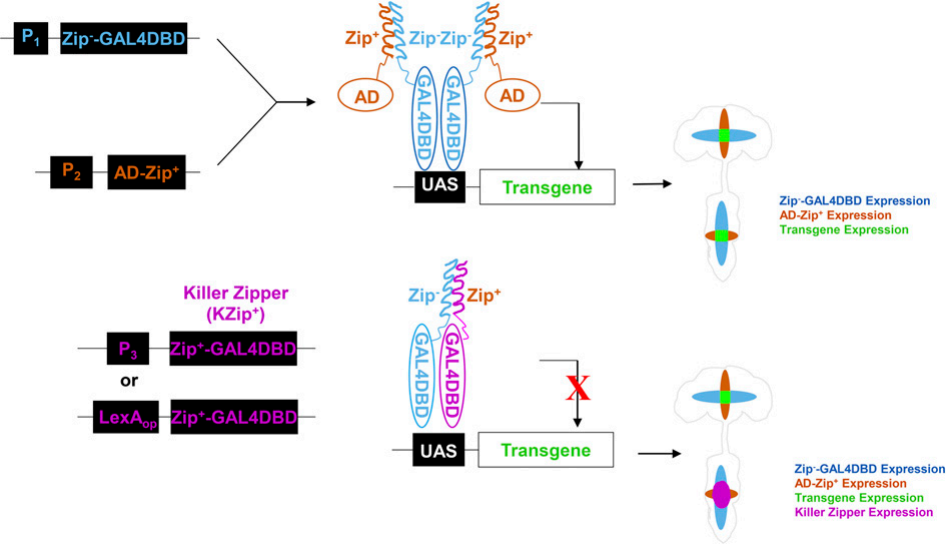
Mechanism of Killer Zipper (KZip^+^) suppression of Split GAL4 activity. (A) The Split GAL4 system consists of functionally distinct transcriptional components, a GAL4 DNA-binding domain (GAL4DBD), and a transcription activation domain (AD), each fused to a heterodimerizing leucine zipper (Zip^−^ or Zip^+^). Each component can be placed under the control of a different promoter (P_1_ or P_2_) resulting in two hemidriver lines, which drive the transcriptional components in different populations of cells. When the hemidrivers are combined, Zip^+^ and Zip^–^ dimerize in cells expressing both components, producing a functional Split GAL4 transcription factor capable of transcribing Upstream Activating Sequence (UAS)-transgenes. (B) KZip^+^ (Zip^+^-GAL4DBD) consists of the GAL4DBD fragment fused to the Zip^+^ leucine zipper and can be expressed either directly under the control of a third promoter (P_3_) or indirectly under such control by a LexA driver (LexA_op_). In cells that express the Split GAL4 components as well as KZip^+^, the latter molecule can form homodimers with the Zip^−^-GAL4DBD and thus titrate GAL4DBD partners for the Zip^+^-AD. Furthermore, the homodimers can compete for binding to UAS sites and block transcription by residual functional Split GAL4 heterodimers.

Two transcriptional activators are commonly used in the Split GAL4 system: p65AD-zip^+^ (Pfeiffer *et al.* 2010) and dVP16AD-zip^+^ (Gao *et al.* 2008), the latter an optimized variant of the VP16AD-zip^+^ construct introduced with the original system (Luan *et al.* 2006). Neither of these activation domains is targeted by a natural inhibitor in the way that GAL80 targets the GAL4AD in yeast (Suster *et al.* 2004), and the GAL4AD itself delivers comparatively weak transcriptional activity in the Split GAL4 system (Luan *et al.* 2006; Diao *et al.* 2015). In the absence of methods for suppressing Split GAL4 activity by targeting the activation domains, we therefore sought a method that acts on the zip^−^-GAL4DBD domain common to all optimized activation domains (ADs). The active GAL4 molecule exists as a homodimer, and paired DNA-binding domains are required for recognition of the UAS. We therefore reasoned that a zip^+^-GAL4DBD construct (*i.e.*, a GAL4DBD fused to the zip^+^ zipper, which is complementary to zip^−^ zipper to which the GAL4DBD is normally fused), would bind the zip^−^-GAL4DBD to promote formation of inactive GAL4DBD homodimers, with the capacity to bind DNA without promoting transcription. In the presence of zip^+^-AD molecules the zip^+^-GAL4DBD would additionally compete with zip^+^-AD for binding to zip^−^-GAL4DBD, and thus attenuate formation of transcriptionally active zip^−^-GAL4DBD-AD-zip^+^ molecules. We call the zip^+^-GAL4DBD molecule the KZip^+^ by virtue of its leucine zipper-mediated neutralization of zip^−^-GAL4DBD’s activity within the Split GAL4 system (Figure 1).

### KZip^+^ efficiently blocks split GAL4-mediated transcription

**Figure 2:**
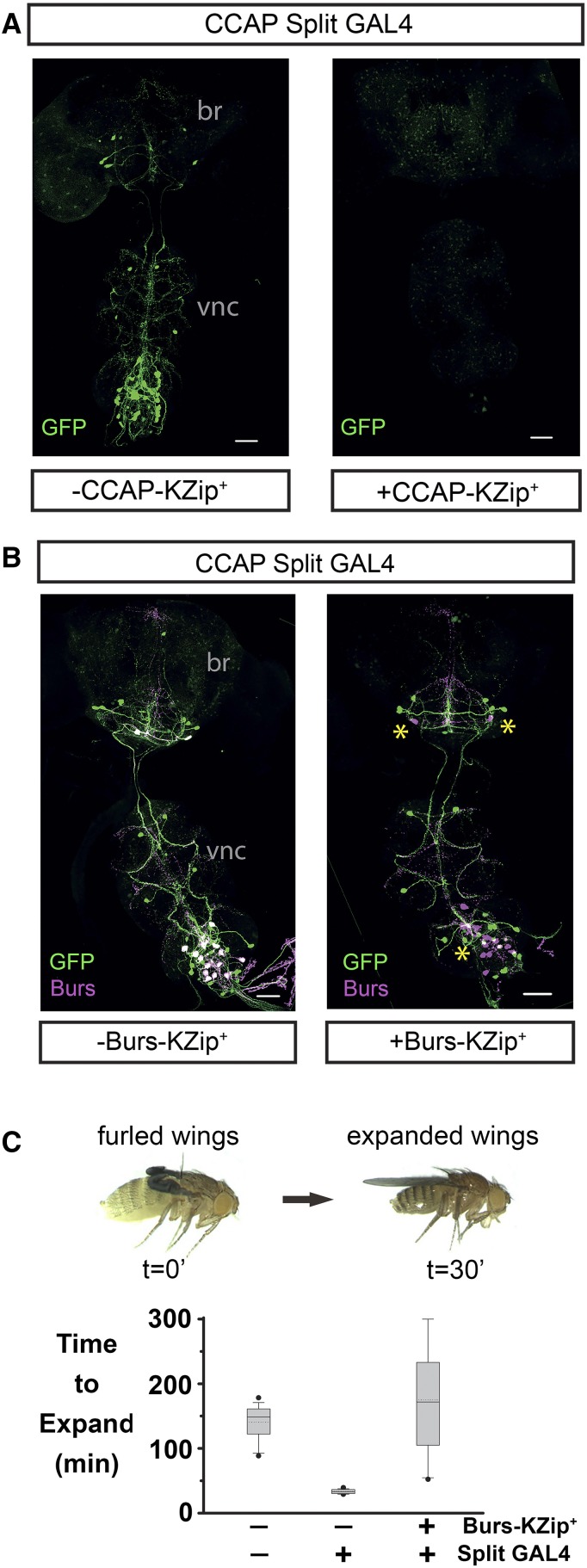
The KZip^+^ construct robustly suppresses Split GAL4-mediated transgene expression. (A) Left: Confocal projection view of a wholemount adult CNS showing the CCAP-expressing neurons visualized by a UAS-2xEGFP reporter (green) driven by a Split GAL4 driver (CCAP-GAL4DBD∩elav-VP16AD). Right: Reporter expression is substantially suppressed when the KZip^+^ is coexpressed in the CCAP neurons. Each image is a representative of *n* = 8 brains per genotype (the genotypes used in the experiments shown in all figures are listed in File S1; here, and in all subsequent figures, UAS-reporter expression has been amplified by anti-GFP immunostaining to stringently test for suppression of Split GAL4 activity by KZip^+^). (B) Left: CCAP neurons expressing 2xEGFP (green) as in (A), but double-labeled with anti-Burs antibody (magenta; double-labeled neurons appear white). Right: Expression of KZip^+^ in the subset of CCAP neurons that coexpresses the *burs* gene selectively blocks 2xEGFP expression in these neurons, which now label only in magenta (asterisks). Bar, 50 μm for all images in (A) and (B). Each image is a representative of *n* = 14 brains per genotype. (C) Top: the wings of newly emerged flies are initially furled and become expanded upon execution of a behavioral program governed by the hormone bursicon. The bursicon-induced program is usually executed within the first 30 min of emergence, but is substantially delayed if flies are confined. Confined flies, however, also expand quickly if the complement of CCAP-expressing neurons that express bursicon is artificially stimulated by activation of the cold-sensitive TRPM8 cation channel. Bottom: Box plots indicate the expansion times of control flies that lack the CCAP-AD hemidriver (left), or flies expressing UAS-TRPM8 in the CCAP-expressing neurons under the control of Split GAL4 (CCAP-AD∩CCAP-DBD > UAS-TRPM8) either with (right) or without (middle) KZip^+^ coexpression in the bursicon neurons. All flies were subjected to a 15 min temperature shift to 18° to activate TRPM8. KZip^+^ expression in the bursicon-expressing neurons prevents their expression of UAS-TRPM8 and therefore their activation by temperature shift. br, brain; Burs, bursicon promoter; CCAP, Crustacean Cardioactive Peptide; EGFP, enhanced GFP; KZip^+^, Killer Zipper; UAS, Upstream Activating Sequence; vnc, ventral nerve cord.

To evaluate the KZip^+^ we first tested inhibition of Split GAL4 activity in a small group of well-characterized neurons that express CCAP. A subset of these cells coexpress the gene encoding the Bursα subunit of the dimeric hormone bursicon and are implicated in wing expansion, a process that concludes metamorphosis (Peabody *et al.* 2009). Using the enhancers for the *CCAP* and *Burs*α genes, we created CCAP- and Bursα-KZip^+^ constructs (Figure S1A) and generated transgenic fly lines by *P*-element-mediated transgenesis. For both constructs, we identified lines that potently suppressed Split GAL4 activity in the targeted neurons (Figure 2). CCAP-KZip^+^ blocked EGFP reporter expression in CCAP-expressing neurons (Figure 2A; note that the genotypes used in all experiments are listed by figure in File S1). Similarly, Bursα-KZip^+^ suppressed Split GAL4 activity in the bursicon-expressing subset of the CCAP neurons, as evidenced by removal of all EGFP expression in the neurons immunopositive for bursicon (Figure 2B). In separate experiments, we tested the ability of the KZip^+^ to inhibit activity of intact GAL4 in bursicon-expressing neurons using the CCAP-GAL4 driver and found substantial but not complete suppression, suggesting that KZip^+^ homodimers may inhibit GAL4 binding to UAS sites (data not shown).

Acute activation of bursicon-expressing neurons with the cold-sensitive ion channel TRPM8 induces rapid wing expansion in recently eclosed adult flies (Luan *et al.* 2012). We tested whether Bursα-KZip^+^ could similarly block this acceleration of wing expansion when UAS-TRPM8 expression was driven in CCAP-expressing neurons by Split GAL4. We found that a single copy of Bursα-KZip^+^ fully blocked the effects of TRPM8-mediated activation of the CCAP neurons (Figure 2C), returning the time to wing expansion to wild-type levels (Luan *et al.* 2012). These data demonstrate the utility of KZip^+^ for functional studies involving Split GAL4-mediated expression of effector transgenes. In addition, our results demonstrate that only those CCAP-expressing neurons that coexpress bursicon can induce wing expansion when activated.

### Optimizing the KZip^+^ construct

The above applications demonstrate the effectiveness of KZip^+^. To determine whether its efficacy might be further improved, we created variants of the KZip^+^ construct designed to express KZip^+^ at elevated levels or to exhibit enhanced transcriptional repression. To achieve the former goal, we incorporated translational enhancers (Pfeiffer *et al.* 2012) into one construct (KZip^+^-p10) and tandem repeats into another (4xKZip^+^), using the T2A peptide (Diao and White 2012); to achieve the latter goal, we fused the repressor domain of the transcriptional regulator *Hairy* (Fisher *et al.* 1996) to the KZip^+^ C-terminus (KZip^+^::HRD). In addition, to facilitate detection of the expressed KZip^+^, we made and tested a construct that contained a C-terminal triplet hemagglutinin tag (KZip^+^::3xHA). To systematically compare the efficacy of these constructs, we tested their performance, together with that of the original KZip^+^ construct, under identical conditions. All constructs (Figure S1B) were placed under the control of the CCAP promoter and inserted into the same genomic locus (attP2 on chromosome III) using ΦC31-mediated integration to control for position effects (Groth *et al.* 2004). Although all KZip^+^ variants substantially attenuated Split GAL4 activity, only the translationally enhanced KZip^+^-p10 construct showed greater efficacy than the original KZip^+^ (Figure S1, C and D). It was particularly surprising that the 4xKZip^+^ construct did not exhibit greater inhibitory potential than KZip^+^ alone, but noteworthy that three of the four KZip^+^ translation products produced by this construct bear a C-terminal T2A-peptide tag (Donnelly *et al.* 2001). Both the KZip^+^::HRD and KZip^+^::3xHA constructs also bear C-terminal fusions, and the reduced efficacy of these constructs relative to KZip^+^ suggests that modifications of the C-terminus may somewhat attenuate KZip^+^ function.

### Generalized, amplified expression of KZip^+^ using the LexA-system

These results demonstrate that KZip^+^ is capable of suppressing Split GAL4 activity when expressed under the direct control of individual gene enhancers. However, this application requires the creation of a new transgenic line for each enhancer, and the expression levels of KZip^+^ are constrained by the transcriptional efficacy of that particular enhancer. To provide a more general means of expressing KZip^+^ in distinct patterns, we made and tested several “universal” constructs (Figure S2A) that can be expressed in arbitrary sets of neurons using the LexA-LexA_op_ system (Lai and Lee 2006). This binary system does not otherwise interfere with expression driven by Split GAL4 (Lai and Lee 2006; Pfeiffer *et al.* 2010) and a large number of well-characterized LexA drivers is now available (http://flystocks.bio.indiana.edu/Browse/lexA/lexA_Janelia.php), thus enabling the expression of KZip^+^ in thousands of different neural populations across the fly brain. We created several different LexA_op_-KZip^+^ constructs, including versions both without (LexA_op_-KZip^+^) and with a 3xHA-tag (LexA_op_-KZip^+^::3xHA) to allow immunohistochemical detection of expression (Figure S2B). Although inclusion of the C-terminal tag was expected to somewhat reduce suppressor activity of KZip^+^ based on our optimization experiments, we reasoned that amplified expression of the construct by the LexA-LexA_op_ system might compensate for this reduction in activity. However, we also created an alternative, untagged construct (LexA_op_-nLacZ-T2A-KZip^+^) that permits visualization of KZip^+^ expression by coupling it to that of nuclear-localized β-galactosidase, which can be detected immunocytochemically or histologically (Figure S2C).

**Figure 3:**
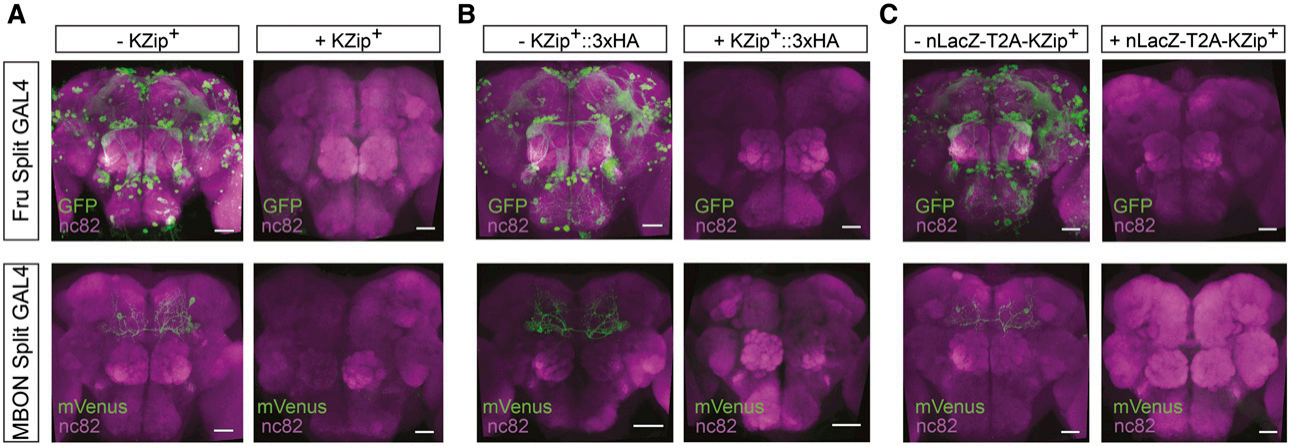
Killer Zipper (KZip^+^) expression driven by LexA drivers potently suppresses Split GAL4 activity. Adult brains from animals expressing Upstream Activating Sequence (UAS)-reporters under the control of Split GAL4 drivers that express in populations of *fru*-expressing neurons (top; JK1029-VP16AD∩Cha-GAL4DBD) or Mushroom Body output neurons (MBON) (bottom; 93D10- p65AD∩13F04-DBD). In both cases, reporter expression (green) is shown in the absence (left) or presence (right) of one of the LexA_op_-KZip^+^ variants driven by the pan-neuronal n-syb-LexA driver (control preparations on the left are from animals missing only the n-syb-LexA transgene, and are otherwise identical in genotype to those from experimental animals shown on the right; each image is representative of *n* > 6 brains per genotype.) Complete suppression of reporter expression is seen with: (A) the LexA_op_-KZip^+^ construct, (B) the LexA_op_- KZip^+^::3xHA construct, and (C) the LexA_op_-LacZ-T2A-KZip^+^ construct. Reporters: UAS-EGFP and UAS-csChrimson::mVenus. Bar, 30 μm.

To test these lines, we selected two strong, previously described Split GAL4 lines: Fru Split GAL4 (Cha-GAL4DBD∩FruJK1029-VP16AD Kohl *et al.* 2013) and a Mushroom Body Output Neuron (MBON)-specific Split GAL4 (R13F04-GAL4DBD∩R93D10-p65AD Aso *et al.* 2014a,b). For both lines, all three LexA_op_-KZip^+^ constructs (including LexA_op_-KZip^+^::3xHA), completely abolished expression of the UAS reporter when expressed in all neurons under the control of a pan-neuronal LexA driver (Figure 3). These results indicate that KZip^+^ retains high efficacy when used with the LexA system. Together, these examples also test all commonly used Split GAL4 DBD and AD components and demonstrate the universal efficacy of the KZip^+^ with the Split GAL4 system.

### Applications of KZip^+^

The Boolean “NOT” operation effected by KZip^+^ on Split GAL4 expression patterns enables two principle applications with respect to a cell group of interest. First, KZip^+^ can remove cells from a Split GAL4 expression pattern that may confound an experiment, leaving only the cells of interest to be selectively labeled or manipulated. Second, KZip^+^ can eliminate the cells of interest from a Split GAL4 expression pattern to determine the effects of their selective exclusion from manipulations performed on the entire pattern. The first application is useful because the probability of finding completely cell type-specific Split GAL4 lines is low. The second application is useful in instances where systematic, functional interrogation of different subpopulations within a Split GAL4 pattern is desirable.

**Figure 4:**
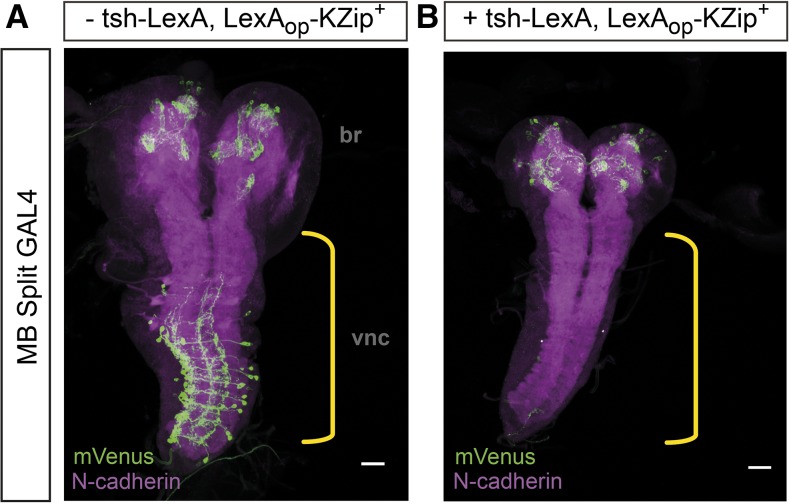
Anatomically parsing neural circuitry with the Killer Zipper (KZip^+^). (A) Confocal projection view of a third-instar larval CNS wholemount expressing Upstream Activating Sequence (UAS)-csChrimson::mVenus (green) under the control of the MB Split GAL4 line (126E12-p65AD∩103H02-DBD). Magenta; neuropil labeling by anti-N-cadherin antibody. Yellow bracket; ventral nerve cord (vnc). br; brain. (B) Confocal image from the CNS of a similar animal additionally expressing LexA_op_- KZip^+^::3xHA under the control of the *teashirt*-LexA driver (tsh-LexA), which expresses primarily in the VNC (yellow bracket). Expression of KZip^+^ suppresses reporter expression in the VNC (genotypes of animals in (A) and (B) were identical except for the presence of the *teashirt*-LexA driver transgene). Bar, 30 μm. Each image is a representative of *n* > 15 brains per genotype.

To illustrate the utility of eliminating unwanted expression from a Split GAL4 pattern, we examined a hemidriver pair that expresses in the larval Mushroom Body (MB Split GAL4; Figure 4A), a brain region which has been the focus of considerable study (Heisenberg 2003). The larval MB expression is coupled with unwanted expression in neurons of the VNC in third-instar larvae. This was eliminated by driving KZip^+^::3xHA under the control of *teashirt*-LexA (J.-M. Knapp and J. Simpson, unpublished data), which expresses predominantly in VNC neurons (Figure 4B). Similarly, by driving LexA_op_-KZip^+^::3xHA under the control of the glial-specific LexA line (Lai and Lee 2006), nonneuronal glia cells were removed from the expression pattern of a Split GAL4 hemidriver pair that otherwise expresses in a single olfactory interneuron of the lateral horn (Figure S3). KZip^+^ can thus remove undesired expression from Split GAL4-generated patterns to substantially reduce the number of cells within the pattern.

**Figure 5:**
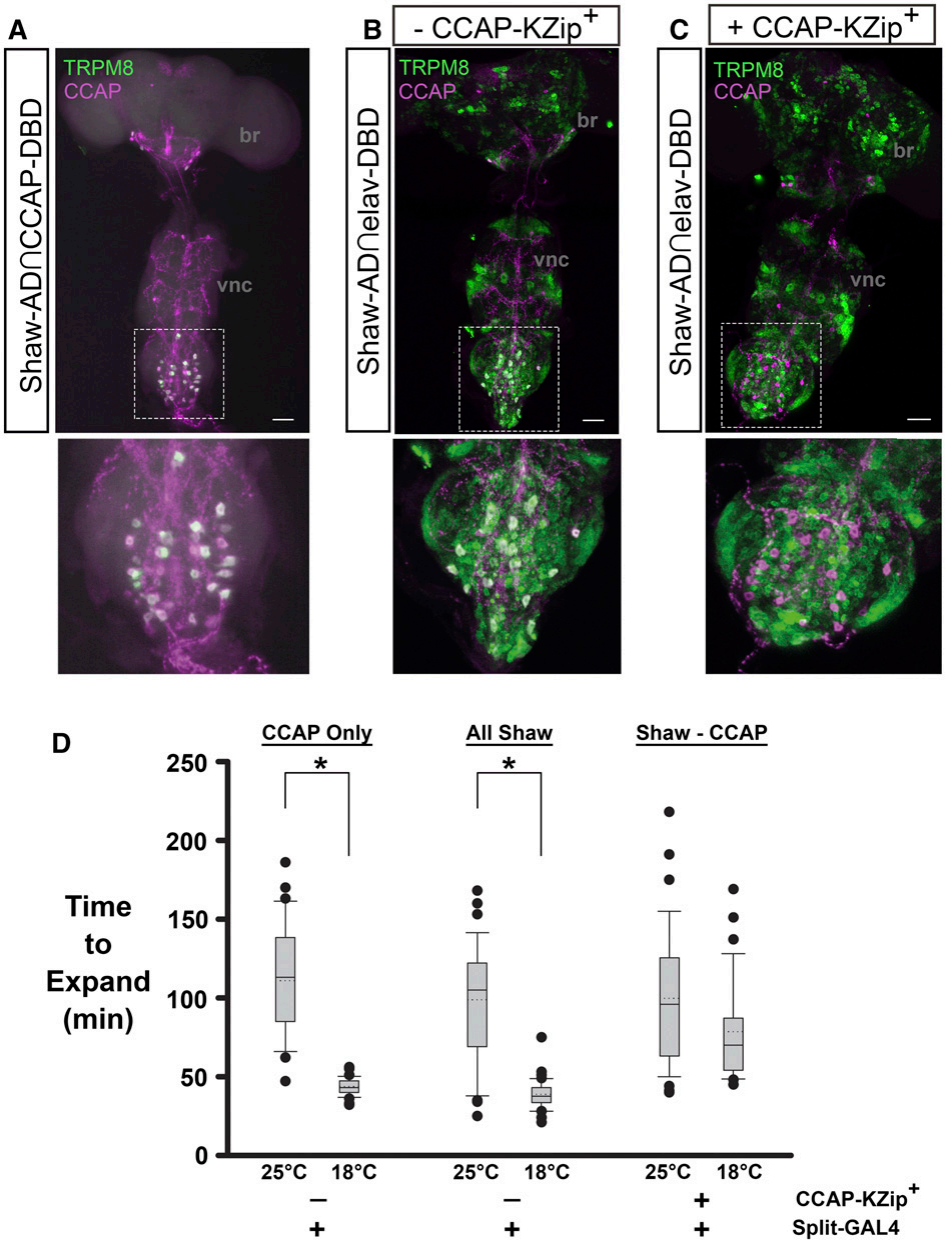
KZip^+^-p10 identifies the subset of Shaw-expressing neurons that promote wing expansion. (A) Top: the *shaw* gene is expressed in a subset of CCAP-expressing neurons identified by the Split GAL4 driver Shaw^MI01735^- p65AD∩CCAP-GAL4DBD driving UAS-EGFP-TRPM8 (green). CCAP neurons are identified by anti-CCAP immunolabeling (magenta) and the double-labeled Shaw-expressing subset appears white. EGFP-TRPM8 expression was detected by anti-GFP immunolabeling and for all panels. Bar, 50 μm. Bottom: Inset of the double-labeled subset within the dotted rectangle in the top panel. (B) Top: the full pattern of *shaw* gene expression in the nervous system—revealed by the Shaw^MI01735^-p65AD∩elav-GAL4DBD Split GAL4 driver—includes not only CCAP-expressing neurons (magenta, with double-labeled neurons appearing white), but also many other neurons as indicated by UAS-EGFP-TRPM8 expression (green). Bottom: Inset as in (A). (C) Top: KZip^+^ expression driven by the CCAP promoter, selectively suppresses Split GAL4 activity in the CCAP neurons, which now do not express the UAS-EGFP-TRPM8 and are labeled only by anti-CCAP (magenta). Bottom: inset as in (A) showing the subset of Shaw- and CCAP-expressing neurons, in which Split GAL4 activity—and therefore TRPM8 expression—is suppressed by KZip^+^. (D) Box plots show the wing expansion times for flies with the genotypes represented in (A)–(C), in which the cold-sensitive ion channel UAS-TRPM8 is expressed in: only CCAP-expressing Shaw neurons (A), all Shaw neurons (B), or all Shaw neurons except those expressing CCAP (C). Flies of the first two types expand rapidly at 18° Compared to 25° by virtue of TRPM8 activation in CCAP-expressing neurons at the lower temperature [asterisks indicate a significant difference in wing expansion time (*P* < 0.05) at the two temperatures as determined by Bonferroni-adjusted Welch’s *t*-tests]. In contrast, flies in which KZip^+^ prevents UAS-TRPM8 expression in CCAP-expressing neurons show no significant difference in wing expansion times at the two temperatures. br, brain; CCAP, Crustacean Cardioactive Peptide; DBD, DNA-binding domain; EGFP, enhanced GFP; KZip^+^, Killer Zipper; UAS, Upstream Activating Sequence; vnc, ventral nerve cord.

As an illustration of the second application of KZip^+^ described above, namely to functionally interrogate neurons within a Split GAL4 expression pattern, we used the KZip^+^-p10 construct to investigate the function of two different subsets of neurons that express the Shaw K^+^ channel. *Shaw* is expressed in CCAP-expressing neurons, including those that express bursicon, and its previously demonstrated importance in wing expansion is likely due, at least in part, to its activity in those neurons (Hodge *et al.* 2005). However, the channel is also expressed in many other neurons (Diao *et al.* 2015) and we sought to determine whether these non-CCAP-expressing neurons might also play a role in wing expansion. We first used the cold-activated ion channel TRPM8 to demonstrate that activation of either all Shaw-expressing neurons (Shaw^MI01735^-p65AD∩elav-GAL4DBD; Figure 5A) or the subpopulation that expresses CCAP (Shaw^MI01735^-p65AD∩CCAP-GAL4DBD; Figure 5B) induces rapid wing expansion, as expected. We then examined the functional consequences of activating the subpopulation of Shaw-expressing neurons that do not express CCAP (Figure 5C). We find that flies in which UAS-TRPM8 expression is specifically blocked in the CCAP-expressing neurons by CCAP-KZip^+^-p10 do not expand their wings significantly more rapidly than control flies. These results indicate that, among Shaw-expressing neurons, those that also express CCAP are primarily, if not solely, responsible for inducing rapid wing expansion and illustrate the utility of KZip^+^ in the functional decomposition of different cell types within an expression pattern.

## Discussion

The Split GAL4 system is widely used in *Drosophila* to map neural circuits, and the KZip^+^ technology introduced here significantly extends the capabilities of that system by providing a method for rationally and reproducibly subdividing the expression patterns of Split GAL4 drivers. It does so by robustly repressing Split GAL4 activity within defined subsets of neurons and is therefore capable of suppressing the expression UAS-transgenes encoding both reporters, such as UAS-EGFP, and effectors, such as UAS-TRPM8. We demonstrate the effectiveness of KZip^+^ both when it is expressed under the control of specific enhancers, such as *CCAP* or *Burs*α, and when its expression is amplified under the control of LexA drivers. The toolbox of KZip^+^ constructs and fly lines presented here should thus be broadly useful in making refined anatomical and functional manipulations to support circuit mapping efforts in *Drosophila*.

The utility of KZip^+^ in restricting the expression pattern of any given Split GAL4 driver will, of course, depend upon the availability of enhancers that can be used to drive its expression in desired cell groups. A distinct advantage of the LexA_op_-KZip^+^ fly lines introduced here is that they can be used with the growing number of publicly available LexA driver lines that express either in functionally defined neuronal groups (*e.g.*, neurons that use a particular neurotransmitter Diao *et al.* 2015; Simpson 2016) or in neuronal patterns that have been extensively characterized and archived in searchable databases so that their likely overlap with a Split GAL4 pattern of interest can be accessed (http://flweb.janelia.org/cgi-bin/flew.cgi). An increasing number of well-characterized Split GAL4 lines is similarly becoming available as part of ongoing efforts at the Janelia Research Campus to generate a set of 4000 Split GAL4 lines that can be used to specifically target transgene expression to most cell types of the fly nervous system (https://www.janelia.org/project-team/flylight), and KZip^+^ should complement these efforts.

Finally, because KZip^+^ acts within the Split GAL4 system analogously to GAL80 within the GAL4-UAS system, tools that exploit GAL80’s repressor function to refine patterns of GAL4-driven transgene expression can be readily adapted for use in the Split GAL4 system. Such tools include FLP-out GAL80 (Struhl and Basler 1993) and FINGR (Bohm *et al.* 2010), both of which have been used successfully for neural circuit mapping (Gordon and Scott 2009; Rezaval *et al.* 2012; Alekseyenko *et al.* 2013; Flood *et al.* 2013; Pool *et al.* 2014). Implementing these strategies with KZip^+^ will, however, necessitate the introduction of additional transgenes into genetic crosses, which may become a limitation.

The KZip^+^ technology introduced here requires the incorporation of up to two transgenes into genetic crosses in addition to the three transgenes required for Split GAL4-mediated expression of a reporter or effector. This number of transgenes is readily accommodated in the fly, and while it may be more challenging to accommodate this number of transgenes in other organisms, ternary expression systems that use the same leucine zippers as Split GAL4 exist in both *Caenorhabditis elegans* (Wei *et al.* 2012) and zebrafish (Almeida and Lyons 2015). Our technology should thus be readily extendable to such organisms. Here, as in flies, achieving sufficiently refined neuronal targeting has been a major limitation, and our hope is that KZip^+^ may broadly facilitate neural circuit mapping studies in multiple genetic model organisms.

## Supplementary Material

Supplemental material is available online at www.genetics.org/lookup/suppl/doi:10.1534/genetics.116.199687/-/DC1.
