# Supplementary material for: Facilitating Neuron-Specific Genetic Manipulations in Drosophila melanogaster Using a Split GAL4 Repressor
Source: Genetics. 2017 Mar 29;206(2):775–84. doi: 10.1534/genetics.116.199687 (PMC5499185; doi:10.1534/genetics.116.199687)
Supplement: Supplementary file 1 [file 775FigureS1.pdf]

Figure S1

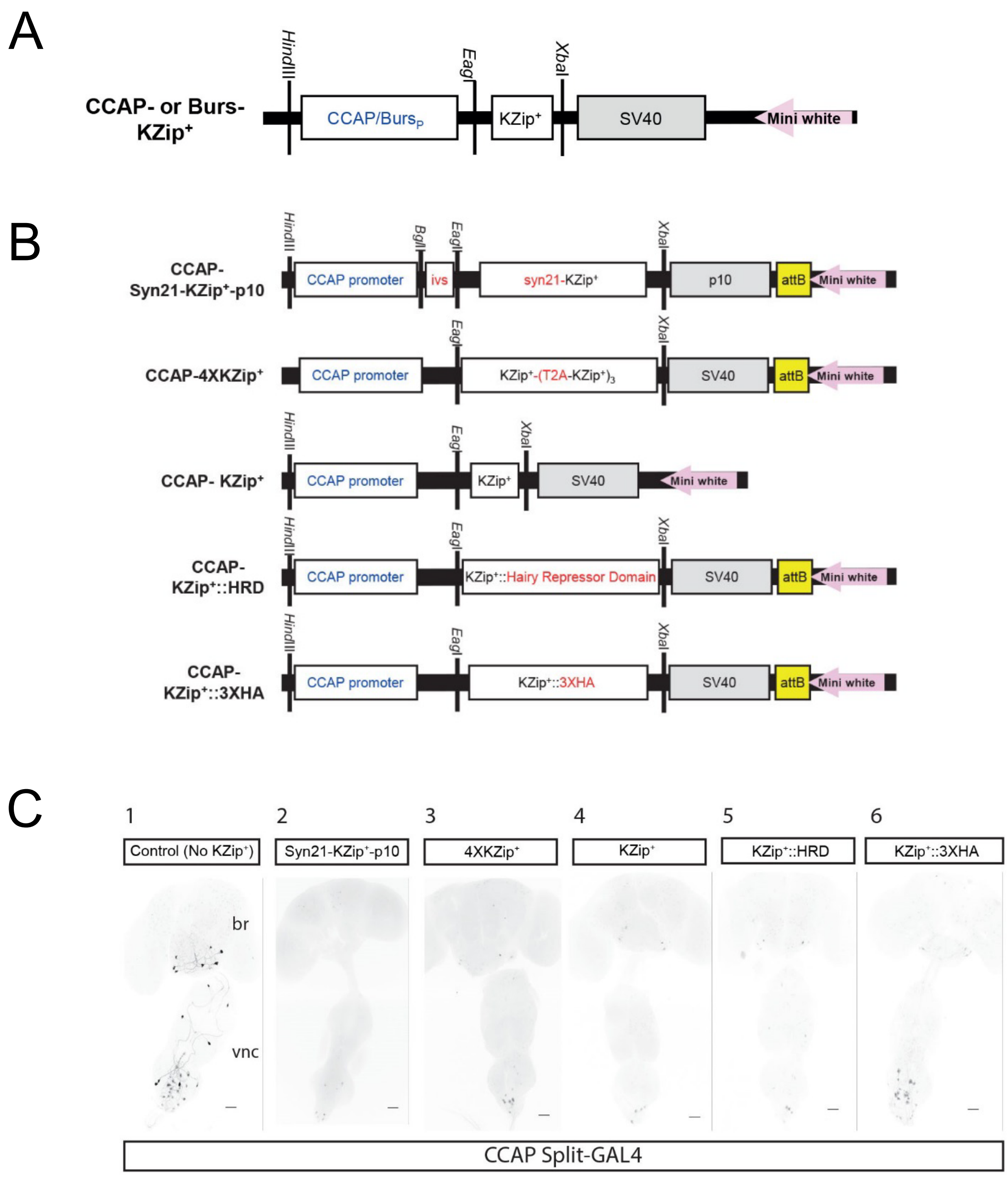

D

Construct	Average # of Cells Labeled	# of Preps	Average Intensity per cell
Control (No KZip$^+$)	31	9	2.3
Syn21-KZip$^+$-p10	8	8	1.0
4XKZip$^+$	20	6	1.5
KZip$^+$	18	6	1.0
KZip$^+$::HRD	17	7	1.1
KZip$^+$-3X::HA	23	6	1.3

Figure S1:

(A) Schematic of the KZip$^+$ constructs designed to be expressed under the control of the *CCAP* or *Burs* gene promoters and used in the experiments shown in Fig. 2A-B.

(B) Schematic of modified KZip$^+$ constructs created to optimize the efficacy of the Killer Zipper technology.

(C) Representative confocal projection views of adult CNS wholemounts expressing a UAS-2XEGFP reporter (black) driven in CCAP-expressing neurons by the elav-VP16AD∩CCAP-GAL4DBD Split GAL4 driver. Each preparation (except for the control, lane 1), additionally expresses the indicated KZip$^+$ construct in CCAP-expressing neurons. KZip$^+$ activity substantially attenuates and/or eliminates reporter expression in all cases with the clearest repression seen with use of translational enhancers (2). All KZip$^+$ constructs containing a C-terminal fusion, including the T2A peptide (3), the Hairy transcriptional repressor domain (5) or the 3XHA tag (6) showed reduced repression compared to the original, untagged KZip$^+$ construct (4).

(D) Quantification of the results for the KZip$^+$ constructs. For each construct tested in (B), confocal Z-stacks of the indicated number of preparations (# of preps) were scored as described in Materials and Methods for the total number of EGFP-labeled CCAP neurons and for the average intensity of labeling per cell (1, lowest level of labeling; 3, highest). The KZip$^+$-p10 construct eliminates reporter expression in approximately 75% of cells and those cells that do show expression are labeled at the lowest level.
