# Supplementary material for: Facilitating Neuron-Specific Genetic Manipulations in Drosophila melanogaster Using a Split GAL4 Repressor
Source: Genetics. 2017 Mar 29;206(2):775–84. doi: 10.1534/genetics.116.199687 (PMC5499185; doi:10.1534/genetics.116.199687)
Supplement: Supplementary file 2 [file 775FigureS2.pdf]

Figure S2

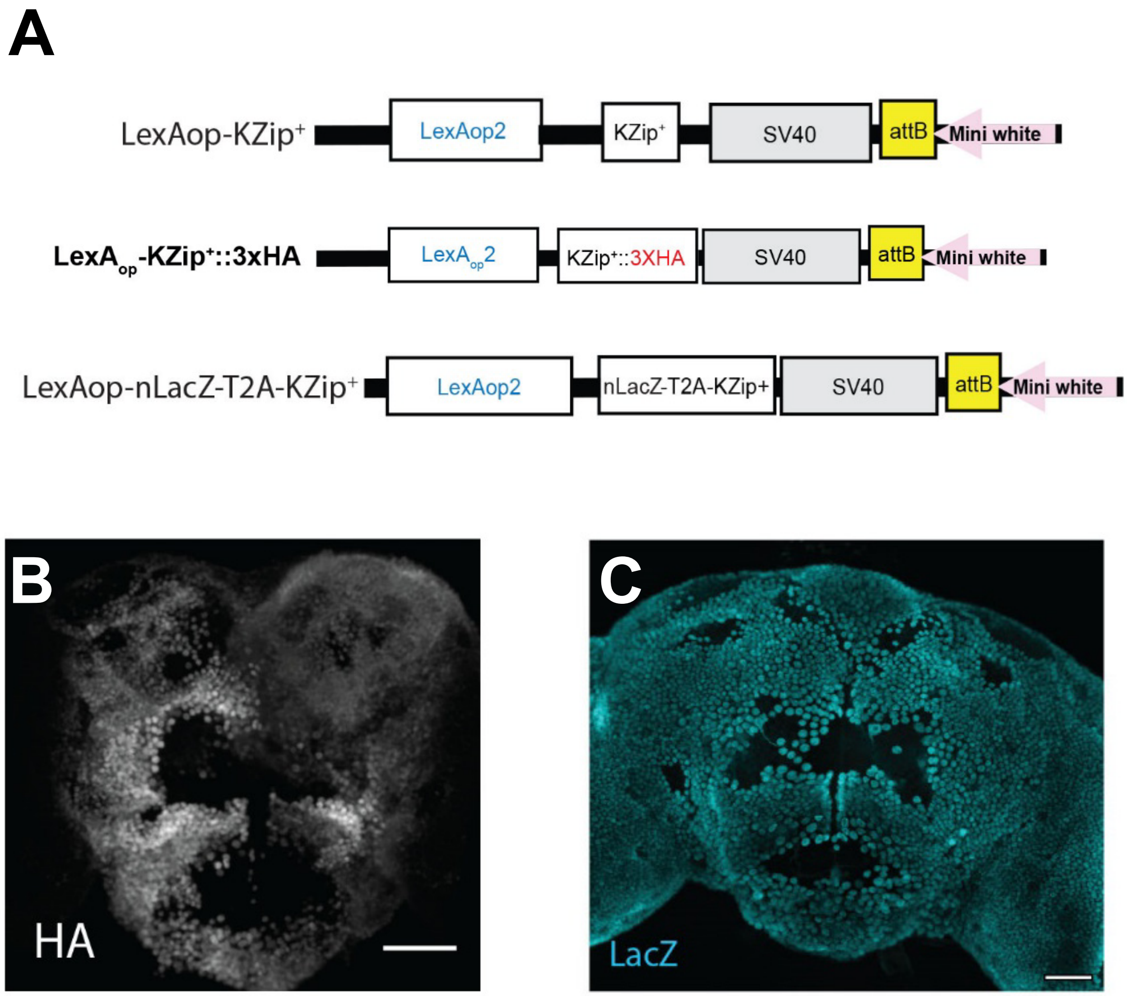

Figure S2:

(A) Schematics of the $LexA_{op}$-$KZip^{+}$ constructs designed to be expressed under the control of LexA drivers. 3XHA, a fusion peptide containing three epitopes recognized by anti-hemeagglutinin antibodies. nLacZ, the gene encoding β-galactosidase fused to a nuclear localization signal. T2A, a viral peptide that promotes ribosomal skipping to generate a separate $KZip^{+}$ protein as described in Materials and Methods.

(B-C) Confocal micrographs of CNS wholemounts showing: (B) anti-HA-immunostaining in an adult brain expressing $KZip^{+}$::3xHA pan-neuronally, or anti-LacZ immunostaining in a brain similarly expressing LexA-LacZ-T2A- $KZip^{+}$.
