# Supplementary material for: Facilitating Neuron-Specific Genetic Manipulations in Drosophila melanogaster Using a Split GAL4 Repressor
Source: Genetics. 2017 Mar 29;206(2):775–84. doi: 10.1534/genetics.116.199687 (PMC5499185; doi:10.1534/genetics.116.199687)
Supplement: Supplementary file 3 [file 775FigureS3.pdf]

Figure S3

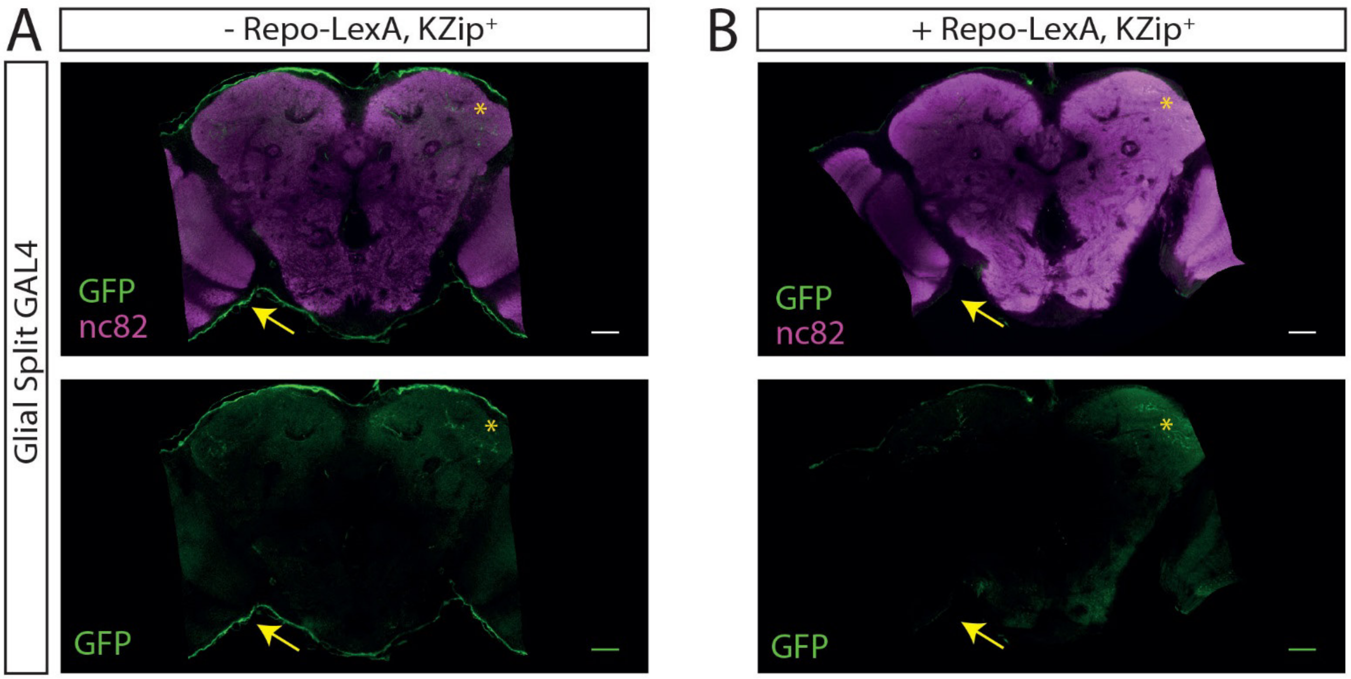

Figure S3:

(A) Confocal images of adult brains showing the expression pattern of Glial Split GAL4 hemidrivers (JK801-AV16AD∩SF131-DBD) driving UAS-GFP (green) and neuropil counterstained with anti-Brp (magenta) in the absence of $KZip^+$ expression. (Bottom panel displays only GFP labeling for clarity). Asterisk, processes of a lateral horn neuron in the otherwise glial pattern. Arrow; glial sheath.

(B) Confocal images of adult brains additionally expressing the $LexA_{op}$-$KZip^+$::3XHA construct in glia using the *repo*-LexA. (Bottom panel displays only GFP labeling for clarity.) Scale bars: 30µm.Schematic of modified $KZip^+$ constructs created to optimize the efficacy of the Killer Zipper technology.
