# Supplementary material for: Facilitating Neuron-Specific Genetic Manipulations in Drosophila melanogaster Using a Split GAL4 Repressor
Source: Genetics. 2017 Mar 29;206(2):775–84. doi: 10.1534/genetics.116.199687 (PMC5499185; doi:10.1534/genetics.116.199687)
Supplement: Supplementary file 4 [file 775FileS1.pdf]

File S1:

Fly Stocks by Figure

Figure 2A:
Control (Left Panel): w/yw; UAS-EGFP/+; elav-VP16AD^{G3A1}, CCAP-GAL4DBD^{K5A1}/+
Experimental (Right panel): w; UAS-EGFP/+; elav-VP16AD^{G3A1}, CCAP-GAL4DBD^{K5A1} /CCAP-KZip^{+13B-1}

Figure 2B:
Control (Left Panel): w; UAS-EGFP/+ ; elav-VP16ADG3A1, CCAP-DBDK5A1/+
Experimental (Right panel): w ; UAS-EGFP/Burs-1xKZip+5 ; elav-VP16ADG3A1, CCAP-DBDK5A1

Figure 2C:
Negative Control (Left): w; Burs-1xKZip+5; CCAP-DBD , UAS-TRPM8 x w; +;+Positive Control (Middle): w; CyO/Sco; CCAP-DBD, UAS-TRPM8/TM6b x w; CCAP-dVP16AD; Dr/TM3
Experimental (Right): w; Burs-1xKZip+; CCAP-DBD , UAS-TRPM8 x w; CCAP-dVP16AD; Dr/TM3

Figure 3A:
Control (Top panel): yw, UAS-mCD8::GFP/Y;UAS-mCD8::GFP/ LexAop- KZip+ (attp40);+/ JK1029-p65AD,Cha-GAL4DBD
Experimental (Top panel): yw, UAS-mCD8::GFP/Y;UAS-mCD8::GFP/ LexAop- KZip+ (attp40);Nsyb-LexA/ JK1029-p65AD,Cha-GAL4DBD
Control (Bottom panel): 20XUAS-csChrimson::mVenus (attp18)/+; LexAop- KZip+ (attp40)/+; 13F04-GAL4DBD,93D10-p65AD /+
Experimental (Bottom panel): 20XUAS-csChrimson::mVenus (attp18)/+; LexAop- KZip+ (attp40)/+; 13F04-GAL4DBD,93D10-p65AD /Nsyb-LexA

Figure 3B:
Control (Top panel): yw, UAS-mCD8::GFP/Y;UAS-mCD8::GFP/LexAop- KZip+::3xHA(attp40);+/JK1029-VP16AD,Cha-GAL4DBD
Experimental (Top panel): yw, UAS-mCD8::GFP/Y;UAS-mCD8::GFP/ LexAop - KZip+::3xHA(attp40);Nsyb-LexA/ JK1029-VP16AD,Cha-GAL4DBD
Control (Bottom panel): 20XUAS-csChrimson::mVenus (attp18)/+; LexAop - KZip+::3xHA(attp40)/+;13F04-GAL4DBD,93D10-p65AD/+
Experimental (Bottom panel): 20XUAS-csChrimson::mVenus (attp18)/+; LexAop - KZip+::3xHA(attp40)/+; 13F04-GAL4DBD,93D10-p65AD/+/Nsyb-LexA

Figure 3C:
Control (Top panel): yw, UAS-mCD8::GFP/Y;UAS-mCD8::GFP/ LexAop-nucLacZ-T2A- KZip+ (attp40);+/JK1029-p65AD,Cha-GAL4DBD
Experimental (Top panel): yw, UAS-mCD8::GFP/Y;UAS-mCD8::GFP/ LexAop-nucLacZ-T2A- KZip+ (attp40);Nsyb-LexA/ JK1029-p65AD,Cha-GAL4DBD
Control (Bottom panel): 20XUAS-csChrimson::mVenus (attp18)/+; LexAop-nucLacZ-T2A- KZip+ (attp40)/+;13F04-GAL4DBD,93D10-p65AD/+
Experimental (Bottom panel): 20XUAS-csChrimson::mVenus (attp18)/+; LexAop-nucLacZ-T2A- KZip+ (attp40)/+; 13F04-GAL4DBD,93D10-p65AD /Nsyb-LexA

Figure 4A:

Control: 20xUAS-csChrimson::mVenus (attp18); 126E12- p65AD/ LexAop - KZip+::3xHA (attp5) ;103H02-GAL4DBD/+

Figure 4B:

Experimental: 20xUAS-csChrimson::mVenus (attp18); 126E12- p65AD/ LexAop - KZip+::3xHA (attp5) , teashirt-LexA;103H02-GAL4DBD/+

Figure 5A:

ShawMI01735-p65AD/+; CCAP-DBD/UAS-TRPM8

Figure 5B:

ShawMI01735-p65AD/+; elav-DBD/UAS-TRPM8

Figure 5C:

ShawMI01735-p65AD/+; elav-DBD/UAS-TRPM8,CCAP-ivs-Syn21-KZip+-p10

Figure S1C:

1: w; UAS-EGFP/+ ; elav-VP16ADG3A1, CCAP-DBDK5A1/+
2: w; UAS-EGFP/+ ; elav-VP16ADG3A1, CCAP-DBDK5A1/CCAP-ivs-Syn21-Kzip+-P10(attP2)
3: w; UAS-EGFP/+ ; elav-VP16ADG3A1, CCAP-DBDK5A1/CCAP-4X-Kzip+(attP2)
4: w; UAS-EGFP/+ ; elav-VP16ADG3A1, CCAP-DBDK5A1/CCAP-Kzip+(attP2)
5: w; UAS-EGFP/+ ; elav-VP16ADG3A1, CCAP-DBDK5A1/CCAP-Kzip+-HRZ(attP2)
6: w; UAS-EGFP/+ ; elav-VP16ADG3A1, CCAP-DBDK5A1/CCAP-KZip+::3XHA(attP2)

Figure S2B:

20XUAS-csChrimson::mVenus (attp18)/+; LexAop- KZip+::3xHA (attp40)/+;13F04-GAL4DBD,93D10-p65AD/Nsyb-LexA

Figure S2C:

20XUAS-csChrimson::mVenus (attp18)/+; LexAop-nuc::LacZ-T2A- KZip+ (attp40)/+; 13F04-GAL4DBD,93D10-p65AD /Nsyb-LexA

Figure S3:

Control: w;UAS-CD8::GFP/ LexAop- KZip+::3xHA(attp40); JK801-VP16AD, SF131-GAL4DBD/+

Experimental: w, Repo-LexA::GAD;UAS-CD8::GFP/ LexAop- KZip+::3xHA(attp40); JK801-VP16AD, SF131-GAL4DBD /+
