# Supplementary material for: Facilitating Neuron-Specific Genetic Manipulations in Drosophila melanogaster Using a Split GAL4 Repressor
Source: Genetics. 2017 Mar 29;206(2):775–84. doi: 10.1534/genetics.116.199687 (PMC5499185; doi:10.1534/genetics.116.199687)
Supplement: Supplementary file 5 [file 775TableS1.pdf]

Table S1: Oligonucleotides used in for KZip$^+$ cloning

Name	Sequence
**pJFRC19/KZip$^+$_F**	CTTTACTTCAGGCGGCCGCGGCTCGAGCAAAACATGCTGGAGATCGAGGCC
**pJFRC19/ KZip$^+$::HA_R**	GTTCCTTCACAAAGATCCTCTAGATTAAGCGTAATCTGGAACGTCATAT GGATAGGATCCTGCATAGTCCGGGACGTCATAGGGATAGCCCGCATAGT CAGGAACATCGTATGGGTACGATACAGTCAACTGTCTTTGACC
**pJFRC19inverse_F**	TCTAGAGGATCTTTGTGAAGGAAC
**pJFRC19inverse_R**	CTCGAGCCGCGGCCGCCTGAAGTAAAG
**pJFRC19_Stop_ KZip$^+$_R**	GGTTCCTTCACAAAGATCCTCTAGATTACGATACAGTCAACTGTCTTTGACC
**pMA-LacZ_F**	CCTAATTCTTATCCTTTACTTCAGGCGG
**pMA- KZip$^+$_R**	GGTTCCTTCACAAAGATCCTCTAGA
